# WHO IS “OLD” IN AN AFFLUENT RETIREMENT COMMUNITY: QUALIFYING STIGMA THROUGH OUTLIER DATA

**DOI:** 10.1093/geroni/igae098.1491

**Published:** 2024-12-31

**Authors:** Alexandra Crampton

**Affiliations:** Marquette University, Milwaukee, Wisconsin, United States

## Abstract

The purpose of this study has been to compare and potentially reconcile professional constructs defining who is an older adult with lived experience of aging into 80+ decades. In this anthropological field research project, the study site is an affluent, Midwestern retirement community in which the average age is 85. Data were primarily collected through participant observation and semi-structured interviews during several data collection periods from 1 week to 2 months between July 2021 and December 2023. Despite optimal access to resources and identities of privilege, findings show how residents confronted “deep old age” as a highly stigmatized experience and social identity. Demographic and other quantitative data were collected from administrative data. These data helped qualify normative responses from research participants, such as a perceived “rash of deaths,” during the Covid-19 pandemic. In addition, qualitative data provided richness of detail and contextualization that numbers could not. In the objective concerns of a facility caring for older adults and the subjective experiences of residents confronting fears of aging, outlier data included examples of coping and social interactions that did not fit professional nor peer expectations of aging as stigmatizing or debilitating. Such examples inspired hope of alternative constructs that transformed ageist fears of age-related change. Residents who inspired included those who were unusually able to accept frailty, falls, and disability without resignation. In dialogue with research participants, “old” age was redefined as risk at any age of “giving up.”

